# Regulation Mechanism of Special Functional Groups Contained in Polymer Molecular Chains on the Tribological Properties of Modified Ti6Al4V

**DOI:** 10.3390/polym15204060

**Published:** 2023-10-12

**Authors:** Mengmeng Liu, Jing Ni, Caixia Zhang, Lihui Wang, Yue Guo, Zhifeng Liu

**Affiliations:** 1 School of Mechanical Engineering, Hangzhou Dianzi University, Hangzhou 310018, China; 42895@hdu.edu.cn (M.L.); nj2000@hdu.edu.cn (J.N.); 222010095@hdu.edu.cn (Y.G.); 2 Institute of Advanced Manufacturing and Intelligent Technology, Beijing University of Technology, Beijing 100124, China; wanglihui@emails.bjut.edu.cn; 3 Key Laboratory of CNC Equipment Reliability, Ministry of Education, Jilin University, Changchun 130012, China

**Keywords:** functional groups of polymers, surface modification, Ti6Al4V, experimental analyses, molecular simulation, tribological properties

## Abstract

Polymer coatings can effectively improve the surface tribological properties of human implant materials, thereby increasing their service life. In this study, poly(vinylsulfonic acid, sodium salt) (PVS), poly(acrylic acid) (PAA) and poly(vinylphosphonic acid) (PVPA) were used to modify Ti6Al4V surfaces. Experimental analyses were combined with molecular simulation to explore the regulation mechanism of special functional groups contained in polymer molecular chains on the tribological properties of modified surfaces. In addition, the bearing capacities and velocity dependence of different polymer modified surfaces during friction were also explored. The PVS coating, due to physical adsorption, can have an anti-friction effect under NaCl solution lubrication, but is not durable under long-term or repeated usage. Both PAA and PVPA molecular chains can form chemical bonds with Ti6Al4V. Phosphate acid groups can firmly bind to the substrate, and the adsorption of salt ions and water molecules can form a hydrated layer on the PVPA coating surface, achieving ultra-low friction and wear. The adsorption of salt ions would aggravate the surface wear of the PAA-modified Ti6Al4V due to the unfirm binding of carboxyl groups to the substrate, resulting in a high friction coefficient. This study can provide effective guidance for the design of modified polymer coatings on metals.

## 1. Introduction

Titanium alloys have high corrosion resistance, a low elastic modulus and good biocompatibility, and they have become the preferred choice of human implant materials [1,2,3,4]. However, these outstanding properties of titanium alloys cannot fully meet the requirements for clinical applications, such as extremely low friction, which is needed at tribological interfaces of living organisms. Surface modification is an effective method to improve the surface properties of materials while maintaining the original excellent properties of materials. Surface modification to improve the surface properties of titanium alloys has received extensive attention from researchers [5,6].

Hydrophilic polymers have good biocompatibility, similar to that of biological macromolecules, and they exhibit excellent frictional properties in salt solutions, becoming important candidates for the surface modification of human implant materials. The tribological properties of hydrophilic polymer coatings in salt solutions are closely related to the special functional groups carried by the molecular chains [7,8]. The special functional groups carried on the polymer molecular chain structure can control the binding method of the molecular chains to the substrates [9] and the interactions with the molecules in the salt solutions [10]. There are two binding methods of polymer molecular chains to substrates, namely physisorption and chemisorption. Consequently, the modification of polymers on metal surfaces can be carried out in one of these two ways [9]. Physisorption relies on weak intermolecular forces between the special functional groups contained in the polymer molecular chain and the substrates. Such a connection would fail under non-ideal conditions due to delamination, desorption or displacement [9,11]. Chemisorption relies on the formation of a stable chemical bond between the special functional groups of the polymer molecular chains through heat treatment or other ways, so as to increase the stability of the modified coatings and improve the tribological characteristics of the modified metal surfaces [12].

The interaction of special functional groups of polymer molecules with molecules in salt solutions is another important factor that affects the tribological properties of polymer-modified surfaces. Liu et al. studied the microscale friction properties between SiO_2_ probes and poly(2-(methacryloy-loxy)-ethyl phosphorylcholine) (PMPC) brushes by atomic force microscopy (AFM). It was proposed that the charged functional groups on PMPC brush molecular chains can attract the surrounding water molecules to form tenacious hydration shells. The excellent fluidity and uniform shear surface of the hydrated shell made the energy dissipation in the friction process very low, so as to obtain an ultra-low friction coefficient [13]. Klein et al. found that the polyethylene-glycol-modified surface can exhibit ultra-low friction in an aqueous solution due to the hydroxyl groups on its side chain, which can produce hydration and osmotic pressure effects [14,15]. Poly(vinylphosphonic acid) (PVPA) was found to be an excellent hydrophilic polymer in our previous studies [16,17]. A large number of phosphate groups contained in PVPA molecular chains can not only promote the formation of a stable three-dimensional skeleton structure of the PVPA coating, but also interact with hydrated salt ions and water molecules in the salt solutions to achieve ultra-low friction [10,18].

Hydrophilic polymers can effectively reduce the friction coefficient and wear of the modified surfaces in the biological environment. If the polymer modification technology is to be applied, several important factors must be studied, such as the method of binding of the coatings to the substrates, the stability of modified coatings in application, the anti-friction mechanisms of the coatings and so on, which are all closely related to special functional groups in polymer molecular chains. In this study, poly(vinylsulfonic acid, sodium salt) (PVS), poly(acrylic acid) (PAA) and poly(vinylphosphonic acid) (PVPA) were selected to modify Ti6Al4V. The regulation mechanism of the functional groups contained in the polymer molecular chains on the tribological properties of the modified surfaces was studied from the aspects of the binding method of functional groups with the substrate, and the interactions between functional groups and molecules in the salt solution. The bearing properties and velocity dependence of the different polymer-modified surfaces during friction were further analyzed. This study can provide theoretical guidance for the optimization of polymer modification technology for medical implant materials.

## 2. Materials and Methods

### 2.1. Materials

PAA (25 wt % in H_2_O), PVS (30 wt % in H_2_O) and PVPA (30 wt % in H_2_O) solutions were all provided by Sigma-Aldrich (St. Louis, MO, USA). NaCl was purchased from J&K Chemicals (Beijing, China). Ti6Al4V (100 mm × 100 mm) foils with a thickness of 1 mm were supplied by Goodfellow, Inc. (Cambridge, UK) and were cut into squares of 10 mm × 10 mm. The cut Ti6Al4V foils were polished using the method of chemical–mechanical polishing to achieve a flat and smooth surface (Ra ≈ 4 nm). The polishing slurry was provided by Southwest Jiaotong University (Chengdu, Sichuan, China). The polished foils were heated in an oven in air at 130 °C for 4 h and used without cleaning. Polytetrafluoroethylene (PTFE) balls with a roughness of approximately 280 nm were purchased from Taobao, Inc. (Hangzhou, China). All reagents mentioned above were used as received.

### 2.2. Preparation of Different Polymer Coatings

PVS, PAA and PVPA coatings were all prepared on Ti6Al4V substrates based on the method of horizontal evaporative self-assembly [17]. First, the Ti6Al4V foils were heated in an oven in air at 140 °C for 8 h to obtain an oxide layer. The pretreated foils were then placed into a PTFE mold horizontally without cleaning. Polymer (PAA, PVS and PVPA) aqueous solutions with a concentration of 0.001 g/mL were injected into the mold. The mold was then heated at the temperature of 50 °C to accelerate the physical adsorption of polymer molecules on Ti6Al4V. PAA and PVS coatings were formed on Ti6Al4V after heating the samples at 120 °C for 24 h [19], while the PVPA coatings were formed on Ti6Al4V after heating the samples at 260 °C for 6 h [17].

### 2.3. Universal Micro-Tribometer for the Evaluation of Tribological Properties

A universal micro-tribometer (UMT-2, Bruker, Germany) was used to characterize the tribological properties. The friction experiments were performed at room temperature in the reciprocating mode with an initial load of 2.5 N. Briefly, a motor underneath the disc controlled the motor pattern of reciprocation and sliding speed. A precise two-dimensional sensor can measure the normal load and frictional force generated during sliding contact simultaneously. The reciprocating frequency and sliding distance were 2 Hz and 3 mm, respectively, corresponding to a sliding speed of 12 mm/s. A PTFE sphere was sampled as the upper friction pair. The bottom friction pair comprised the bare Ti6Al4V, the PVS-modified Ti6Al4V, the PAA-modified Ti6Al4V and the PVPA-modified Ti6Al4V, successively. The NaCl solution with a concentration of 0.5 mol/L was used as a lubricant. The lower friction pair was completely immersed in the NaCl solution during the friction process. All experimental results were obtained by averaging the values of at least four repetitions.

### 2.4. Surface Characterization

The surface elements of polymer-modified Ti6Al4V were determined by X-ray photoelectron spectroscopy (ESCALAB 250 XI, Thermo Scientific Instrument, Waltham, MA, USA) equipped with a monochromatized Al K R X-ray source. The scan step was 0.1 eV. All spectra were obtained at a 90° photoelectron take-off angle from the surface. All binding energy values were charge-referenced to the C1s hydrocarbon peak at 284.8 eV. At least three points were analyzed on each measured surface.

A contact angle measuring instrument (DATA PHYSICS, DCAT21, Filderstadt, Germany) was used to measure the contact angles of deionized water on different surfaces. This was used to evaluate the hydrophilic and hydrophobic properties of the surfaces. During the test, deionized water (2 µL) was added to the surface to be tested, and the contact angle value was tested after stabilizing for 1 s. Three points were measured on each sample, and the contact angle value was the average of nine points in the three samples.

The surface morphologies of the scratches after sliding were observed using a stereo light microscope (BX53M, OLYMPUS, Tokyo, Japan). Before each test, the samples were washed using deionized water. The magnification of sample surfaces was 100 or 200 times.

The micro-morphologies of soaked surfaces and wear marks were observed by field-emission scanning electron microscopy (Gemini SEM 300, Carl Zeiss Aktiengesellschaft, Oberkochen, Germany). Energy-dispersive spectroscopy (EDS) and high-angle annular dark-field (HAADF) images were captured by an aberration-corrected scanning transmission electron microscope operated at 30 kV to obtain the surface element distribution of scratches after sliding.

### 2.5. Density Functional Theory (DFT) Study

Density functional theory (DFT) calculations were performed to determine the interaction energy of different molecules and ions on the polymer system. The simulations were performed using the DMol3 code in Materials Studio (2019) [20]. The physical wave functions were expanded in terms of the Dmol3/GGA-PBE/DNP (3.5) numerical basis set [21]. The core electrons were treated with DFT semi-core pseudopotentials [22]. The exchange–correlation energy was calculated using the Perdew–Burke–Ernzerhof (PBE) formulation of the generalized gradient approximation (GGA) [23]. A Fermi smearing of 0.005 Ha (1 Ha = 627.5 Kcal/mol) and a global orbital cutoff of 5.2 Å were employed. The convergence criteria for the geometric optimization and energy calculation were set as follows: (a) a self-consistent field tolerance of 1.0 × 10^−6^ Ha/atom; (b) an energy tolerance of 1.0 × 10^−5^ Ha/atom; (c) a maximum force tolerance of 0.002 Ha/Å; (d) a maximum displacement tolerance of 0.005 Å.

## 3. Results and Discussion

### 3.1. Stability of Modified Polymer Coatings

**Figure 1:**
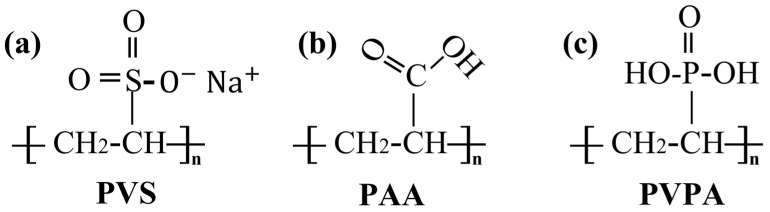
Chemical structures of polymer molecular chains: (**a**) PVS; (**b**) PAA; (**c**) PVPA.

The Ti6Al4V surfaces were modified by poly(vinylsulfonic acid, sodium salt) (PVS), poly(acrylic acid) (PAA) and poly(vinylphosphonic acid) (PVPA). The special functional groups contained in these three polymer molecular chains are the sodium sulfonate group, carboxy group and phosphate group. The chemical structures of the polymers are illustrated in Figure 1.

**Figure 2:**
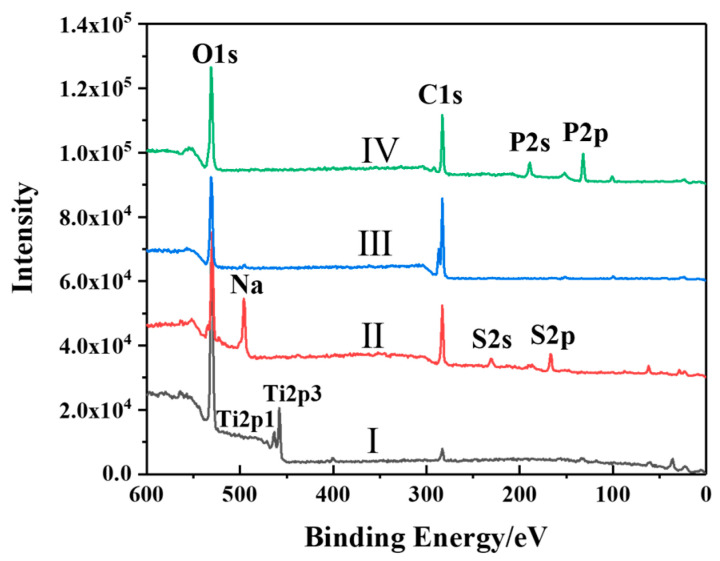
×PS scans of (I) bare Ti6Al4V and (II–IV) polymer-modified Ti6Al4V (II: PVS; Ⅲ: PAA; Ⅳ: PVPA).

The surface information of Ti6Al4V before and after being modified by different polymers was analyzed using XPS. Figure 2 presents typical XPS survey scans over a binding energy in the range of 0 to 600 eV. Line I in Figure 2 represents the typical XPS survey of the Ti6Al4V surface before modification. Lines II, III and IV indicate the typical XPS surveys of the surfaces of PVS-modified Ti6Al4V, PAA-modified Ti6Al4V and PVPA-modified Ti6Al4V, respectively. To illustrate a stark contrast, the ordinate values were successively shifted upwards by 30,000 from the second line. Compared with Line I, the disappearance of titanium peaks and the increase in carbon peaks in Line II, III and IV confirmed that the Ti6Al4V surfaces were successfully modified. However, the appearance of Na and S peaks in Line II and P peak in Line IV proved the existence of PVS and PVPA molecules on the Ti6Al4V surface.

**Figure 3:**
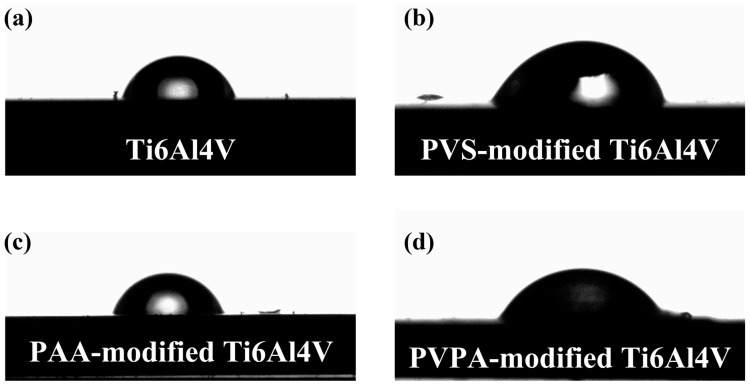
The contact angles of the deionized water on surfaces: (**a**) Ti6Al4V; (**b**) PVS-modified Ti6Al4V; (**c**) PAA-modified Ti6Al4V and (**d**) PVPA-modified Ti6Al4V.

The contact angles of the deionized water on surfaces were further tested to determine the hydrophilic and hydrophobic characteristics of the surfaces. The results can be seen in Figure 3. By taking an average of several measurements, the contact angles of deionized water on a bare Ti6Al4V surface, PVS-modified Ti6Al4V surface, PAA-modified Ti6Al4V surface and PVPA-modified surface were 76°, 70°, 75° and 50°, respectively. This indicates that PVS, PAA and PVPA are all hydrophilic polymers, and the modification of these three coatings on Ti6Al4V does not change the hydrophilic properties of Ti6Al4V. It is worth noting that the contact angle of deionized water on the PVPA-modified Ti6Al4V surface was significantly lower than that of the bare Ti6Al4V surface, which proves that the hydrophilic property of the PVPA-modified surface is significantly improved.

**Figure 4:**
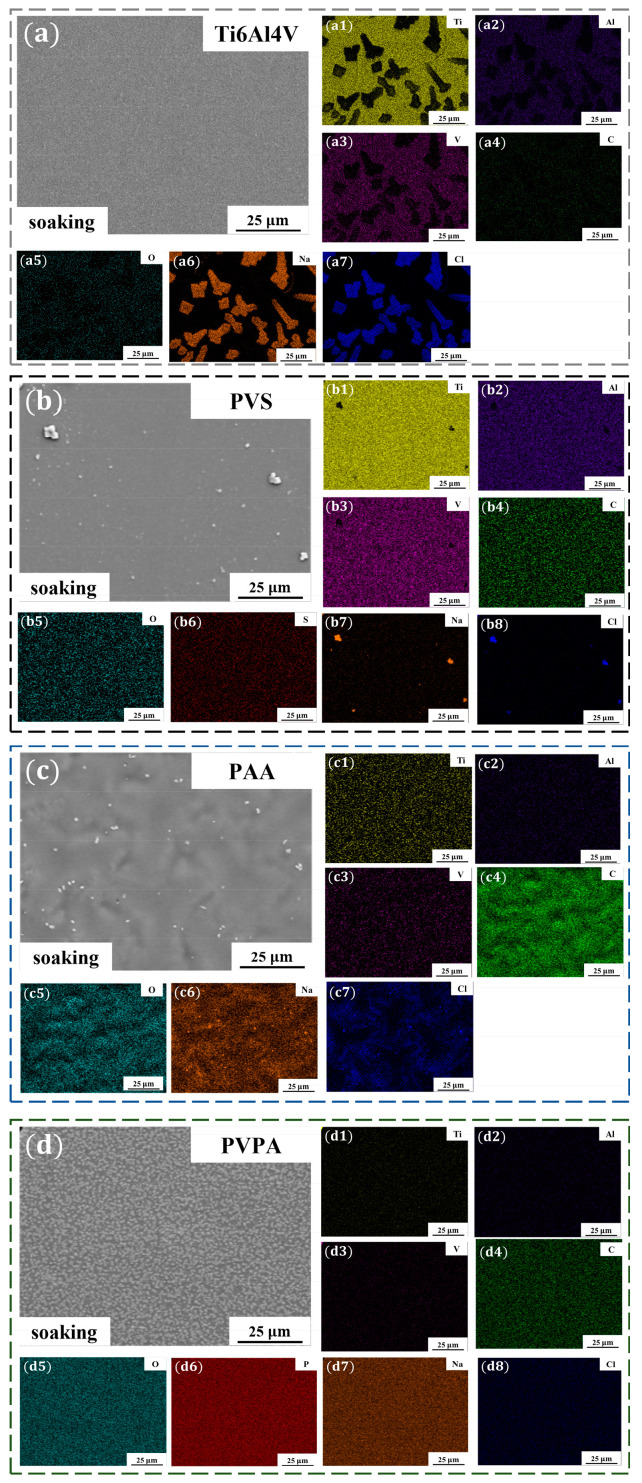
(**a**) Morphology of the soaked bare Ti6Al4V; (**a1**–**a7**) EDS mapping in the region of (**a**); (**b**) morphology of the soaked PVS-modified Ti6Al4V; (**b1**–**b8**) EDS mapping in the region of (**b**); (**c**) morphology of the soaked PAA-modified Ti6Al4V; (**c1**–**c7**) EDS mapping in the region of (**c**); (**d**) morphology of the soaked PVPA-modified Ti6Al4V; (**d1**–**d8**) EDS mapping in the region of (**d**).

The stability of polymer coatings on Ti6Al4V in the salt solution was analyzed to lay a foundation for studying the regulation mechanism of polymer functional groups on the tribological properties of modified surfaces. The bare Ti6Al4V, PVS-modified Ti6Al4V, PAA-modified Ti6Al4V and PVPA-modified Ti6Al4V were soaked in the NaCl solution with a concentration of 0.5 mol/L for 30 min, and then removed and dried for use. The surface morphologies and element distributions of soaked samples were detected by SEM and EDS, respectively. Figure 4a shows the surface topography of the bare Ti6Al4V after soaking in the NaCl solution, and the distribution of elements on the bare Ti6Al4V surface can be seen in Figure 4(a1–a7). Figure 4b–d show the surface morphologies of the soaked PVS-modified Ti6Al4V, the PAA-modified Ti6Al4V and the PVPA-modified Ti6Al4V, respectively. The element distributions of corresponding surfaces are shown in Figure 4(b1–b8,c1–c7,d1–d8). The element distribution results are recorded in Table 1.

Bare Ti6Al4V contains five basic elements (Ti, Al, V, C and O); after soaking in the NaCl solution, the surface mass ratios of these five elements were 86.55 ± 0.38%, 5.57 ± 0.06%, 3.42 ± 0.12%, 1.56 ± 0.11% and 2.91 ± 0.39%, respectively. The elements of Na and Cl could hardly be detected on the soaked bare Ti6Al4V surface. It can be considered that Na^+^ and Cl^−^ hardly adsorbed on the bare Ti6Al4V surface. For the soaked PVS-modified Ti6Al4V, the mass ratio of S was 0 + 0.03%. The mass ratio of C on the soaked PVS-modified Ti6Al4V surface was 3.67 ± 0.10%, which was larger than that on the soaked bare Ti6Al4V. It can be considered that part of the mass ratio of C detected on the soaked PVS-modified surface came from the substrate and the other part came from the PVS molecular chain. In other words, there were still some PVS molecules on the surface after immersing in the NaCl solution. The elements of Na and Cl could be detected on the soaked PVS-modified surface, and the mass ratios were 0.36 ± 0.03% and 0.26 ± 0.02%, respectively. Since Na^+^ and Cl^−^ were almost non-adsorbed on the bare Ti6Al4V surface, the detection of Na and Cl elements can be attributed to the contribution of PVS molecular chains. For the soaked PAA-modified Ti6Al4V surface, the elements with high surface mass ratios were C (58.65 ± 0.17%), O (22.58 ± 0.16%), Na (10.41 ± 0.06%) and Cl (6.82 ± 0.05%). The basic elements of Al and V in the Ti6Al4V substrate were hardly detected, while the mass ratio of Ti on the surface was only 1.54 ± 0.05%. It can be considered that the PAA coating on the Ti6Al4V surface can stably exist in the NaCl solution, and the detected elements of C and O were from the PAA coating. The presence of Na and Cl with higher mass ratios on the coating surface indicates that Na^+^ and Cl^−^ can adsorb on the PAA molecular chain effectively. The elements of C, O and P occupied large mass ratios on the soaked PVPA-modified Ti6Al4V surface, and the specific values were 24.44 ± 0.20%, 27.01 ± 0.11% and 21.67± 0.08%, respectively. The mass ratio of the inherent element Ti was 1.6 ± 0.03%, and the elements of Al and V were almost absent, proving the stable existence of the PVPA coating in the NaCl solution. The elements of Na (18.03 ± 0.06%) and Cl (7.25 ± 0.04%) occupied larger mass ratios on the soaked PVPA-modified surface, which means that the PVPA molecular chain was able to attract Na^+^ and Cl^−^ effectively as well.

**Figure 5:**
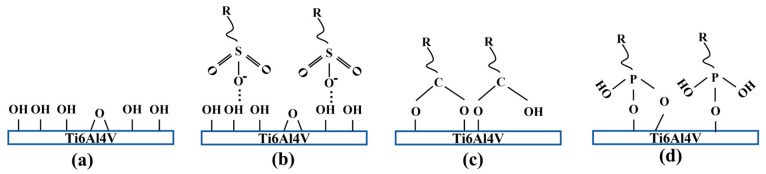
(**a**) Heat−treated Ti6Al4V surface; (**b**) reaction of sulfonate groups with heat-treated Ti6Al4V surface; (**c**) reaction of carboxy groups with heat-treated Ti6Al4V surface; (**d**) reaction of phosphate groups with heat-treated Ti6Al4V surface.

The special functional groups of polymer molecular chains can affect the stability of the modified coatings in the NaCl solution. The stability of polymer coatings has to do with the binding method of functional groups to the substrates. In this study, Ti6Al4V foils were heat treated before modification, and there were large hydroxyl groups (OH) on the surface, as shown in Figure 5a. The PVS coating on Ti6Al4V cannot exist stably in the NaCl solution. It can be speculated that there was no stable chemical bond between the PVS molecular chain and the Ti6Al4V substrate. Combined with the chemical structure shown in Figure 1a, it was believed that sulfonic groups in the PVS molecular chains were physically adsorbed on the Ti6Al4V surface by electrostatic force, as shown in Figure 5b. Norio Nakayama et al. used carboxylic acid and long-chain alkyl amine to modify the TiO_2_ surface, reducing the severe aggregation of nanoparticles in the process of preparing nanocomposite films. It has been proved that the carboxylic group can form chemical bonds with Ti-OH of the TiO_2_ surface [24]. Combined with the stability of the PAA coating in the NaCl solution, carboxyl groups contained in PAA molecular chains can be considered to form stable chemical bonds with the Ti-OH on the Ti6Al4V surface during the modification process, as shown in Figure 5c. Previous studies have showed that PVPA molecules can be connected to the Ti6Al4V substrate through P-O-Ti covalent bonds, as shown in Figure 5d, ensuring the stability of the entire PVPA coating on Ti6Al4V [17].

### 3.2. Analysis of the Regulation Mechanism of Polymer Functional Groups on the Tribological Properties of Modified Surfaces

The tribological properties of polymer-modified surfaces can effectively reflect the properties of the polymers and the modification effect. The tribological properties of Ti6Al4V before and after the modification of PVS, PAA and PVPA were analyzed in this section.

**Figure 6:**
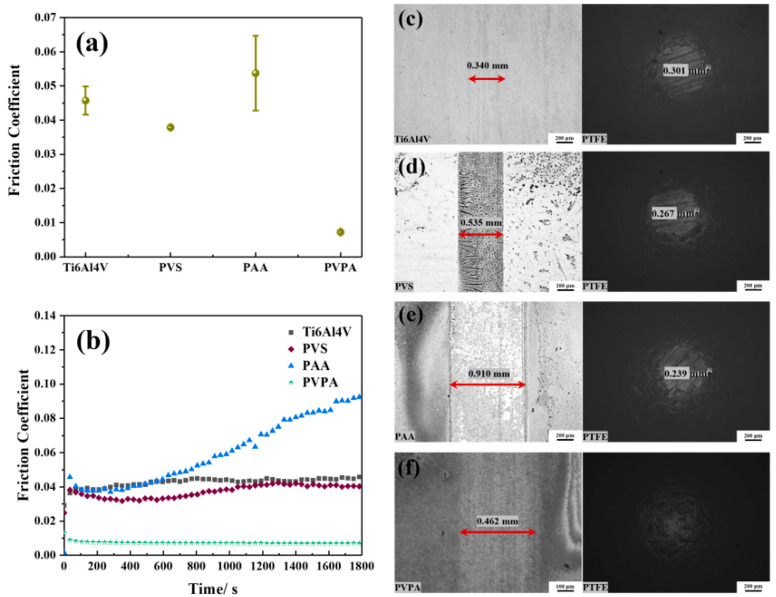
Tribological properties of the bare and polymer-modified Ti6Al4V under NaCl solution lubrication: (**a**) the average friction coefficient; (**b**) the friction coefficient versus time; (**c**) wear morphologies of the bare Ti6Al4V and the PTFE ball; (**d**) wear morphologies of the PVS-modified Ti6Al4V and the PTFE ball; (**e**) wear morphologies of the PAA-modified Ti6Al4V and the PTFE ball; (**f**) wear morphologies of the PVPA-modified Ti6Al4V and the PTFE ball.

The friction coefficients of the bare Ti6Al4V, PVS-modified Ti6Al4V, PAA-modified Ti6Al4V and PVPA-modified Ti6Al4V were 0.0457 ± 0.0041, 0.0379 ± 0.0005, 0.0537 ± 0.0109 and 0.0072 ± 0.0005, respectively, as shown in Figure 6a. Compared with the bare Ti6Al4V, the friction coefficient of the PVS- and PVPA-modified Ti6Al4V decreased. PVPA-modified Ti6Al4V even achieved an ultra-low friction state. However, the friction coefficient of the PAA-modified Ti6Al4V increased. Figure 6b shows the variation trend in the bare Ti6Al4V, PVS-modified Ti6Al4V, PAA-modified Ti6Al4V and PVPA-modified Ti6Al4V with time. The friction coefficient of the PVS-modified Ti6Al4V surface fluctuated obviously with time, which was similar to that of the bare Ti6Al4V. As is shown in Figure 5c,d, the wear widths of the two surfaces were similar as well, but an obvious salt ion adsorption phenomenon appeared on the wear surface of PVS-modified Ti6Al4V. The wear cross-sectional area of the PTFE ball rubbed against the PVS-modified Ti6Al4V and was 0.267 mm^2^, which was smaller than that of the PTFE ball, which rubbed against the bare Ti6Al4V (0.301 mm^2^). The friction coefficient of PAA-modified Ti6Al4V increased significantly with time, and the wear marks of the modified surface and the PTFE ball can be seen in Figure 6e. Compared with the bare Ti6Al4V and PVS-modified Ti6Al4V, the wear width of the PAA-modified Ti6Al4V surface was larger, but the wear cross-sectional area of PTFE was smaller. The friction coefficient of PVPA-modified Ti6Al4V was very stable within the sliding time (30 min). The wears of this modified surface and the corresponding PTFE ball were very shallow, which can be ignored, as shown in Figure 6f.

Figure S1 in the Supporting Information shows the results of a 4 h friction test. Compared with the friction time of 30 min (Figure 6), the friction coefficient of the bare Ti6Al4V surface and the wear marks of the friction pairs were significantly increased. The friction coefficients of PVS-modified Ti6Al4V, PAA-modified Ti6Al4V and PVPA-modified Ti6Al4V surfaces and the corresponding wears of friction pairs also increased and intensified with the increase in friction time. However, the PVPA-modified Ti6Al4V surface was always in an ultra-low friction state during the friction process. The trends in friction coefficient of the PAA-modified Ti6Al4V surface and the wear marks of friction pairs over time were also smaller than that of the PVS-modified Ti6Al4V. Therefore, it can be considered that PVPA is the most durable of the three modified coatings, followed by PAA and PVS.

**Figure 7:**
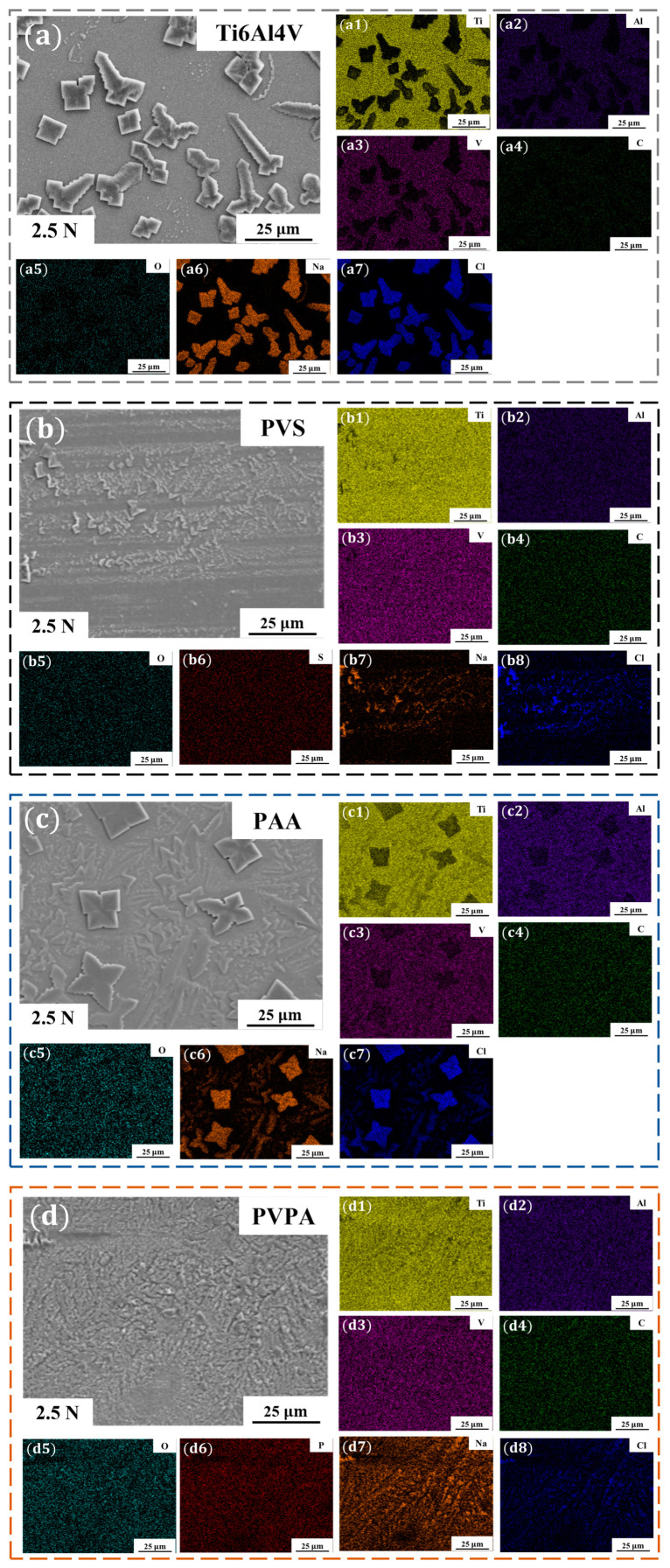
(**a**) Morphology of the bare Ti6Al4V wear area; (**a1**–**a7**) EDS mapping in the region of (**a**); (**b**) morphology of the soaked PVS-modified Ti6Al4V; (**b1**–**b8**) EDS mapping in the region of (**b**); (**c**) morphology of the soaked PAA-modified Ti6Al4V; (**c1**–**c7**) EDS mapping in the region of (**c**); (**d**) morphology of the soaked PVPA-modified Ti6Al4V; (**d1**–**d8**) EDS mapping in the region of (**d**).

SEM and EDS were used to analyze surface morphologies and chemical element compositions of the bare Ti6Al4V, PVS-modified Ti6Al4V, PAA-modified Ti6Al4V and PVPA-modified Ti6Al4V wear marks. Figure 7a shows the surface topography of the wear mark of the bare Ti6Al4V. The distribution of elements in Figure 7a are recorded in Figure 7(a1–a7). Figure 7b–d shows the surface morphologies of the wear marks of the PVS-modified Ti6Al4V, the PAA-modified Ti6Al4V and the PVPA-modified Ti6Al4V. The element distributions of Figure 7b–d are shown in Figure 7(b1–b8,c1–c7,d1–d8). The element distribution results are recorded in Table 2.

The surface mass ratios of Ti, Al, V, C, O, Na and Cl of the bare Ti6Al4V wear mark were 65.18 ± 0.19%, 4.03 ± 0.04%, 2.74 ± 0.08%, 2.43 ± 0.14%, 1.61 ± 0.21%, 13.07 ± 0.07% and 10.94 ± 0.06%, respectively. Combined with the distribution of elements on the soaked bare Ti6Al4V surface, it can be believed that Na^+^ and Cl^−^ can be adsorbed on the bare Ti6Al4V surface due to the shear action during friction. For the wear mark of PVS-modified Ti6Al4V, the mass ratios of Ti, Al, V, C, O, S, Na and Cl were 82.23 ± 0.23%, 5.35 ± 0.04%, 3.72 ± 0.08%, 3.67 ± 0.10%, 2.55 ± 0.23%, 0 + 0.03%, 1.44 ± 0.04% and 1.03 ± 0.02%, respectively. Compared with the surface element distribution of soaked PVS-modified Ti6Al4V, the mass ratios of Na and Cl increased, while the mass ratio of Ti decreased, which means that the shear action during friction can also increase the adsorption of Na^+^ and Cl^−^ on the PVS-modified Ti6Al4V surface. The mass ratios of the remaining elements were almost constant, indicating that the PVS coating was durable in the 30 min friction experiment. To determine the durability of the PVS coating under the condition of multiple usage, three repeated friction experiments were performed on the same area of the PVS-modified Ti6Al4V surface, the results of which are recorded in Figure S2 of the Supporting Information. The average friction coefficients corresponding to the first, the second and the third friction tests were 0.0379, 0.0434 and 0.0513, respectively, showing a gradually increasing trend. After the first, second and third friction tests, the wear marks of PVS-modified Ti6Al4V showed a gradually increasing trend. The corresponding PTFE friction pair also had the same trend of increasing wear. It can be assumed that the anti-friction effect of the PVS coating will be weakened under multiple uses. For the wear mark of PAA-modified Ti6Al4V, the mass ratios of Ti, Al, V, C, O, Na and Cl were 75.80 ± 0.19%, 4.82 ± 0.03%, 3.41 ± 0.07%, 4.63 ± 0.11%, 1.78 ± 0.19%, 5.34 ± 0.04% and 4.23 ± 0.03%, respectively. Compared with the surface element distribution of soaked PAA-modified Ti6Al4V, the mass ratios of C and O were significantly reduced, and the mass ratios of the Ti, Al and V elements were significantly increased, which means that the PAA coating was obviously damaged under shear action. The surface mass ratios of Ti, Al, V, C, O, P, Na and Cl of the PVPA-modified Ti6Al4V wear mark were 52.95 ± 0.17%, 3.13 ± 0.03%, 1.88 ± 0.07%, 10.67 ± 0.11%, 14.10 ± 0.18%, 5.45 ± 0.03%, 7.46 ± 0.05% and 4.36 ± 0.03%, respectively. Compared with the soaked PVPA-modified Ti6Al4V, the mass ratios of P, C and O were also reduced under shear action, but there were still large mass ratios, which means that the PVPA coating was damaged to a certain extent during friction, but it could still exist stably.

The interactions between the coatings and the external environment, mainly investigated through molecular simulation, were analyzed to determine the reason for the difference in tribological properties of Ti6Al4V modified by different polymers.

**Figure 8:**
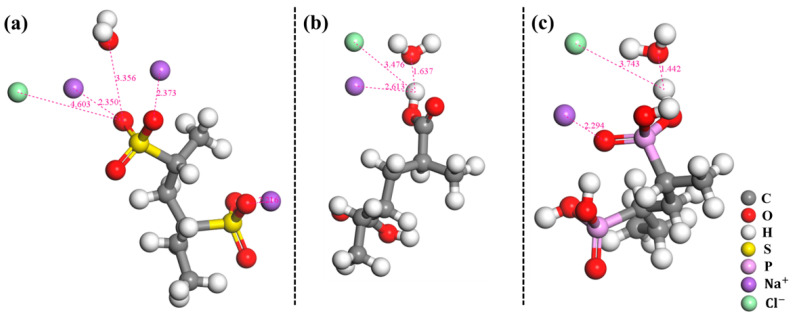
Interaction energy calculation models of (**a**) PAA, (**b**) PVS and (**c**) PVPA.

The external environment of the polymer coatings in the friction process was the NaCl solution, and the components contained in the system models should include the polymer molecular chain, the water molecule, Na^+^ and Cl^−^. Interactions between polymer molecular chains and the NaCl solution mainly included interactions with Na^+^, Cl^−^ and the water molecule, which could be analyzed by DFT calculations. An amorphous MS module was used to construct the molecular models of PVS, PAA, PVPA and water, as well as the ion models of Na^+^ and Cl^−^. The polymerization degrees of PAA, PVS and PVPA molecular chains were all *n* = 2. The Forcite module was used to optimize these models. The polymer coating surface was simplified to some extent when constructing the system mode. One Na^+^, one Cl^−^ and one optimized water molecule were placed on the optimized polymer molecular chain to form the system model. The adsorptions of salt ions and water molecules on the polymer molecular chain were approximated to their adsorptions on the polymer coating surface. DFT calculation with the Dmol3/GGA-PBE/DNP basis set was performed to optimize the convergence of energy charge, displacement and force. The interaction models of DFT-optimized PVS, PAA and PVPA molecular chains with salt ions and surrounding water molecules are shown in Figure 8a, b and c, respectively. It can be seen that the shortest distances between salt ions and the water molecule to polymer molecular chains were their distances to the special functional groups of the polymer molecular chains. Therefore, it can be considered that salt ions and water molecules mainly interact with special functional groups of polymer molecular chains.

The interaction energy (E_int_) indicating the strength of the interaction between the components in the system can be calculated as follows:

(1) $$E_{\mathrm{int}}{=E}_{\mathrm{tot}}-E_{\mathrm{com}}$$

where E_tot_ and E_com_ are the total energy of the system and the energy of its component, respectively. A negative E_int_ value corresponds to stable adsorption of the components and a more negative value of E_int_ indicates a stronger interaction in the system. The interaction energies between polymer molecular chains and salt ions as well as the water molecule were investigated. The results are recorded in Table 3.

The interaction energies of the PVS molecular chain with Cl^−^ and the water molecule were 0.21 Kcal/mol and 1.08 Kcal/mol, respectively. Positive interaction energies indicated that the PVS molecular chain has a certain repulsive effect on Cl^−^ and the water molecule. The interaction energy of the PVS molecular chain with Na^+^ was −33.31 Kcal/mol. Compared with that of PAA (−3.23 Kcal/mol) and PVPA (−6.33 Kcal/mol), the attraction degree of the PVS molecular chain to Na^+^ was significantly increased. This was mainly due to the Na^+^ contained in the PVS molecular chain, resulting in the increase in the overall number of Na^+^. Na^+^ adsorbed on the functional groups of PVS molecules can attract the surrounding water molecules to form a hydrated layer, thus reducing friction. For the polymer coatings that were covalently bonded to the Ti6Al4V (PAA and PVPA), the interaction energies of PAA molecular chains with Na^+^, Cl^−^ and the water molecule were −3.23 Kcal/mol, −1.77 Kcal/mol and −8.90 Kcal/mol, respectively. The interaction energies of the PVPA molecular chain with Na^+^, Cl^−^ and the water molecule were −6.33 Kcal/mol, −3.09 Kcal/mol and −14.91 Kcal/mol, respectively. The negative interaction energy indicated that Na^+^, Cl^−^ and the water molecule can stably adsorb on the PAA and PVPA molecular chains. Water molecules adsorbed on the special functional groups of polymer molecular chains can form a hydration layer. However, the adsorbed Na^+^ and Cl^−^ can attract surrounding water molecules as well, promoting the formation of a hydrated layer on the polymer coating surface, thereby reducing friction. The absolute values of the interaction energies of the PVPA with salt ions and the water molecule were greater than that of PAA, which indicated that the attraction degree of PVPA to salt ions and the water molecule were greater than that of PAA. This was an important reason why the friction coefficient of PVPA-modified Ti6Al4V was smaller than that of PAA-modified Ti6Al4V.

The results of molecular simulation show that both PAA and PVPA molecular chains can attract salt ions and water molecules very effectively. However, the PAA-modified Ti6Al4V exhibited a higher friction coefficient and surface wear (30 min friction time). Analysis of the element distribution in the wear mark of PAA-modified Ti6Al4V (Figure 7) shows that the PAA coating was seriously damaged after the friction experiment. Although both PAA and PVPA molecular chain are chemically bound to the Ti6Al4V substrate, carboxyl groups of the PAA molecular chain are not firmly bound to the substrate, and salt ions in NaCl solution will accelerate the surface wear during friction.

**Figure 9:**
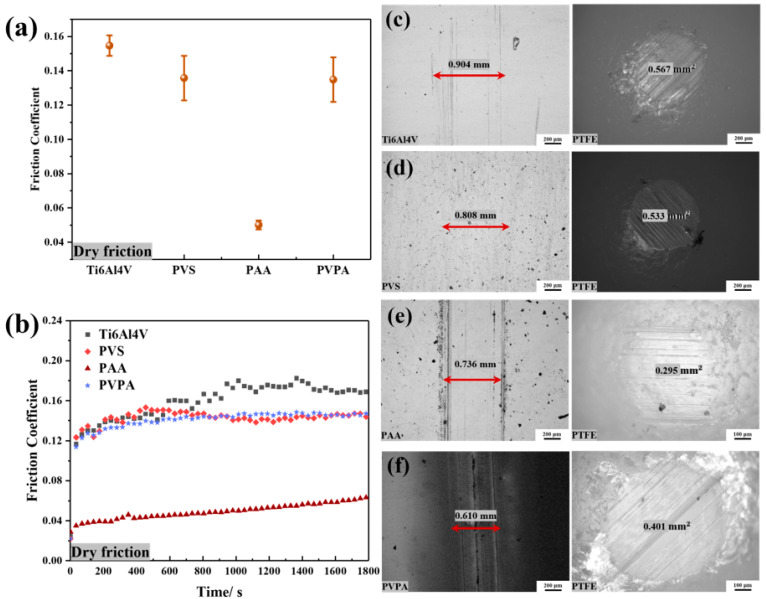
Tribological properties of the bare and polymer-modified Ti6Al4V under dry friction: (**a**) the average friction coefficient; (**b**) the friction coefficient versus time; (**c**) wear morphologies of the bare Ti6Al4V and the PTFE ball; (**d**) wear morphologies of the PVS-modified Ti6Al4V and the PTFE ball; (**e**) wear morphologies of the PAA-modified Ti6Al4V and the PTFE ball; (**f**) wear morphologies of the PVPA-modified Ti6Al4V and the PTFE ball.

To verify this conjecture, the same friction parameters as in the friction experiment in Figure 6 were maintained. Moreover, the bare Ti6Al4V, the PVS-modified Ti6Al4V, the PAA-modified Ti6Al4V and the PVPA-modified Ti6Al4V were subjected to dry friction experiments, successively. The result can be seen in Figure 9. Under dry friction, the friction coefficients of the bare Ti6Al4V, PVS-modified Ti6Al4V, PAA-modified Ti6Al4V and PVPA-modified Ti6Al4V were 0.1547 ± 0.0059, 0.1358 ± 0.0130, 0.0501 ± 0.0026 and 0.1349 ± 0.0130, respectively, as shown in Figure 9a. The change in friction coefficient over time under dry friction can be seen in Figure 9b. Compared with the NaCl solution lubrication, the friction coefficients of the bare Ti6Al4V, PVS-modified Ti6Al4V and PVPA-modified Ti6Al4V were significantly increased under dry friction. This indicates that the NaCl solution can effectively lubricate the bare Ti6Al4V, PVS-modified Ti6Al4V and PVPA-modified Ti6Al4V surfaces during the friction process. It is worth noting that the friction coefficient of the PAA-modified Ti6Al4V under dry friction was smaller than that with lubrication in NaCl solution. The wear marks of the PAA-modified Ti6Al4V and the PTFE ball after dry friction were also similar to those after NaCl solution lubrication, as shown in Figure 6e and Figure 9e. This indicates that the NaCl solution does not provide effective lubrication in the friction process of PAA-modified Ti6Al4V.

The interactions between polymer coatings and the environment can affect the tribological properties of polymer-modified Ti6Al4V. The strong attraction of the sulfonic acid group on the PVS molecular chain to Na^+^ can contribute to effective lubrication in the friction process. However, the PVS coating physically adsorbed on Ti6Al4V is not durable. The anti-friction effect of PVS cannot be durable in the long-term or with repeated usage. Both PAA and PVPA molecular chains can form chemical bonds with Ti6Al4V. However, the functional group contained in the polymer affects the binding strength of the molecular chain to the substrate. Phosphate acid groups on the PVPA molecular chain can firmly bind to the substrate. In addition, phosphate acid groups can effectively attract salt ions and water molecules in the NaCl solution, and the hydrated layer formed on the coating surface enables PVPA-modified Ti6Al4V to obtain an ultra-low friction coefficient and wear. When the binding between functional groups and the substrate is not strong enough, the salt ions in the NaCl solution will accelerate the wear of the polymer-modified surface during the friction process.

### 3.3. Bearing Capacity and Velocity Dependence of Polymer-Modified Ti6Al4V

The bearing capacities and velocity dependence of the bare/polymer-modified Ti6Al4V were analyzed in this section to verify the regulation mechanism of special functional groups on the tribological properties of the polymer-modified Ti6Al4V.

**Figure 10:**
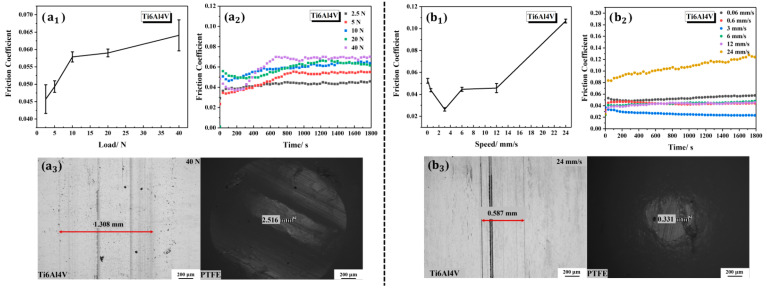
Tribological properties of the bare Ti6Al4V under different loads and at different velocities: (**a_1_**) the average friction coefficients under different loads; (**a_2_**) the friction coefficients versus time under different loads; (**a_3_**) wear morphologies of the bare Ti6Al4V and the PTFE ball under the load of 40 N; (**b_1_**) the average friction coefficients under different velocities; (**b_2_**) the friction coefficients versus time under different velocities; (**b_3_**) wear morphologies of the bare Ti6Al4V and the PTFE ball at the velocity of 24 mm/s.

The bearing capacity and velocity dependence of the bare Ti6Al4V were firstly studied to establish a comparative model for the polymer-modified Ti6Al4V. The friction bearing experiments on the bare Ti6Al4V were carried out with an average speed of 12 mm/s under different loads of 2.5 N, 5 N, 10 N, 20 N and 40 N, successively. The above parameters were also used in the experimental studies on the bearing capacities of polymer-modified Ti6Al4V. The friction coefficients under the loads of 2.5 N, 5 N, 10 N, 20 N and 40 N were 0.0457 ± 0.0041, 0.0493 ± 0.0017, 0.0579 ± 0.0014, 0.0590 ± 0.0011 and 0.0641 ± 0.0045, respectively, as shown in Figure 10(a_1_). It can be seen that the friction coefficient of the bare Ti6Al4V was greatly affected by the load. The larger the applied load, the greater the friction coefficient. It can be seen from Figure 10(a_2_) that the friction coefficients of the bare Ti6Al4V all fluctuated significantly with the change in time under different loads, especially under the applied load of 40 N. Combined with Figure 6c and Figure 10(a_3_), it can be seen that the larger the load, the more serious the wear mark on the bare Ti6Al4V and the PTFE ball.

When studying the influence of velocity on the tribological characteristics of the bare Ti6Al4V, the applied load was maintained at 2.5 N, and other experimental conditions remained unchanged. The reciprocating frequencies were adjusted to 0.01 Hz, 0.1 Hz, 0.5 Hz, 1 Hz, 2 Hz and 4 Hz, successively, corresponding to the velocities of 0.06 mm/s, 0.6 mm/s, 3 mm/s, 6 mm/s, 12 mm/s and 24 mm/s, respectively. The results can be seen in Figure 10b. These parameters were simultaneously used in the experimental studies on the velocity dependence of polymer-modified Ti6Al4V. The friction coefficients under the six gradient velocities of 0.06 mm/s, 0.6 mm/s, 3 mm/s, 6 mm/s, 12 mm/s and 24 mm/s were 0.0524 ± 0.0022, 0.0438 ± 0.0014, 0.02633 ± 0.0015, 0.0446 ± 0.0018, 0.0457 ± 0.0041 and 0.1069 ± 0.00175, respectively, as shown in Figure 10(b_1_). The friction coefficient of the bare Ti6Al4V was significantly affected by the velocity, especially at the low (0.06 mm/s) and high velocities (24 mm/s). Wear marks of the bare Ti6Al4V and the PTFE ball at the speed of 24 mm/s can be seen in Figure 10(b_3_). Figure 10(b_2_) shows the variation trend in the friction coefficient of the bare Ti6Al4V with the change in time at different velocities. At the velocity of 24 mm/s, the friction coefficient fluctuated significantly and tended to increase significantly with the increase in friction time.

**Figure 11:**
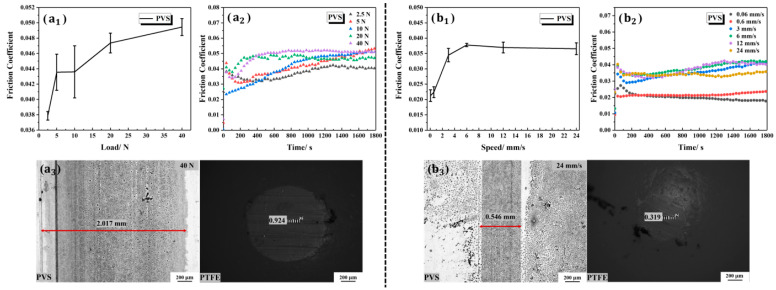
Tribological properties of the PVS-modified Ti6Al4V under different loads and at different velocities: (**a_1_**) the average friction coefficients under different loads; (**a_2_**) the friction coefficients versus time under different loads; (**a_3_**) wear morphologies of the PVS-modified Ti6Al4V and the PTFE ball under the load of 40 N; (**b_1_**) the average friction coefficients under different velocities; (**b_2_**) the friction coefficients versus time under different velocities; (**b_3_**) wear morphologies of the PVS-modified Ti6Al4V and the PTFE ball at the velocity of 24 mm/s.

The average friction coefficients and the variation trend in friction coefficients with the change in time of the PVS-modified Ti6Al4V under different loads are shown in Figure 11(a_1_,a_2_). The wear marks of the PVS-modified Ti6Al4V and the PTFE ball under the load of 40 N can be seen in Figure 11(a_3_). The friction coefficients under the five gradient loads were 0.0379 ± 0.0005, 0.0436 ± 0.0024, 0.0436 ± 0.0034, 0.0474 ± 0.0013 and 0.0495 ± 0.0011. The friction coefficients showed an upward trend with the increase in applied loads. The friction coefficient of the PVS-modified Ti6Al4V was smaller than that of the bare Ti6Al4V under the same load. Its variation range of the friction coefficients under gradient loads was also smaller than that of the bare Ti6Al4V. Combined with Figure 6d and Figure 11(a_3_), it can be found that the variation trend of wear marks with load of PVS-modified Ti6Al4V and its corresponding PTFE ball are similar to that of the bare Ti6Al4V and its corresponding PTFE ball. In other words, using PVS to modify Ti6Al4V can reduce the surface friction coefficient and improve the bearing characteristics of the surface.

Figure 11(b_1_,b_2_) show the average friction coefficient and the variation trend in friction coefficient with time of PVS-modified Ti6Al4V at different velocities. The friction coefficients at the velocities of 0.06 mm/s, 0.6 mm/s, 3 mm/s, 6 mm/s, 12 mm/s and 24 mm/s were 0.0213 ± 0.0019, 0.0224 ± 0.0018, 0.0345 ± 0.0022, 0.0378 ± 0.0005, 0.0370 ± 0.0018 and 0.0365 ± 0.0019, respectively. Compared with bare Ti6Al4V, the velocity dependence of the friction coefficient of PVS-modified Ti6Al4V significantly decreased. The wear marks of the friction pairs at the velocity of 24 mm/s can be seen in Figure 11(b_3_).

**Figure 12:**
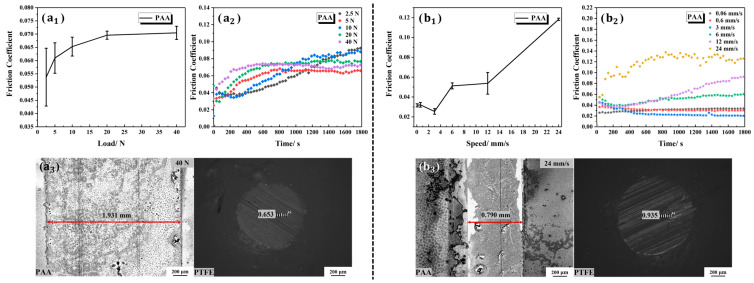
Tribological properties of the PAA-modified Ti6Al4V under different loads and at different velocities: (**a_1_**) the average friction coefficients under different loads; (**a_2_**) the friction coefficients versus time under different loads; (**a_3_**) wear morphologies of the PAA-modified Ti6Al4V and the PTFE ball under the load of 40 N; (**b_1_**) the average friction coefficients under different velocities; (**b_2_**) the friction coefficients versus time under different velocities; (**b_3_**) wear morphologies of the PAA-modified Ti6Al4V and the PTFE ball at the velocity of 24 mm/s.

The tribological characteristics of the PAA-modified Ti6Al4V surface were further analyzed. Figure 12(a_1_,a_2_) show the average friction coefficient and the variation trend in friction coefficient with time, respectively, of the PAA-modified Ti6Al4V under different loads. Under the five gradient loads, the average friction coefficients were 0.0537 ± 0.0109, 0.0610 ± 0.0058, 0.0653 ± 0.0036, 0.0696 ± 0.0015 and 0.0705 ± 0.0025. The friction coefficients of the PAA-modified Ti6Al4V showed a slow rising trend under the five gradient loads, which was similar to that of bare Ti6Al4V. Notably, the friction coefficient of the PAA-modified Ti6Al4V was slightly higher than that of the bare Ti6Al4V under any same load. The surface wear marks under the loads of 2.5 N and 40 N are shown in Figure 6e and Figure 12(a_3_), respectively. It can be seen that the PAA coating was severely scratched under both low and high loads, which was an important reason for the high friction coefficient and weak bearing capacity of the PAA-modified Ti6Al4V.

The friction coefficients of the PAA-modified Ti6Al4V under the six gradient velocities of 0.06 mm/s, 0.6 mm/s, 3 mm/s, 6 mm/s, 12 mm/s and 24 mm/s were 0.0315 ± 0.0016, 0.0321 ± 0.0023, 0.0254 ± 0.0029, 0.0512 ± 0.0030, 0.0537 ± 0.0109 and 0.1182 ± 0.0012, respectively, as shown in Figure 12(b_1_). The friction coefficient of the PAA-modified Ti6Al4V was significantly higher at the velocity of 24 mm/s and fluctuated significantly with the change in time (Figure 12(b_2_)). The corresponding wear marks of the PAA-modified Ti6Al4V and the PTFE ball can be seen in Figure 12(b_3_).

**Figure 13:**
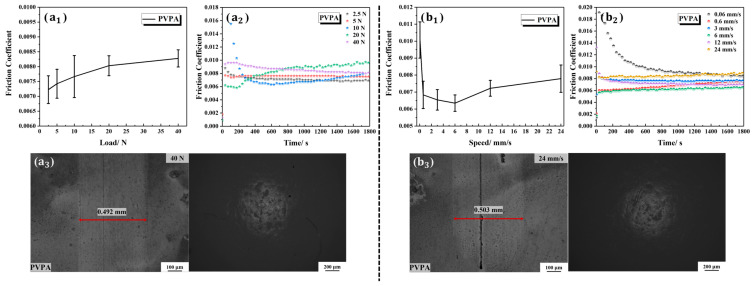
Tribological properties of the PVPA-modified Ti6Al4V under different loads and at different velocities: (**a_1_**) the average friction coefficients under different loads; (**a_2_**) the friction coefficients versus time under different loads; (**a_3_**) wear morphologies of the PVPA-modified Ti6Al4V and the PTFE ball under the load of 40 N; (**b_1_**) the average friction coefficients under different velocities; (**b_2_**) the friction coefficients versus time under different velocities; (**b_3_**) wear morphologies of the PVPA-modified Ti6Al4V and the PTFE ball at the velocity of 24 mm/s.

The tribological properties of the PVPA-modified Ti6Al4V are shown in Figure 13. The friction coefficients obtained under the loads of 2.5 N, 5 N, 10 N, 20 N and 40 N were all less than 0.01, as shown in Figure 13(a_1_). Except for the initial run-in stage, the friction coefficients under the five gradient loads did not fluctuate significantly with time, as shown in Figure 13(a_2_). There were no obvious wear marks on the PVPA-modified Ti6Al4V and the PTFE ball under the load of 40 N, as shown in Figure 13(a_3_). Compared with PAA, the PVPA-modified Ti6Al4V surface had a significantly higher bearing capacity.

The influence of velocity on the tribological characteristics of PVPA-modified Ti6Al4V was further analyzed. At velocities ranging from 0.06 mm/s to 24 mm/s, the PVPA-modified Ti6Al4V all achieved ultra-low friction (Figure 13(b_1_)), and the friction coefficients did not fluctuate significantly with the change in time (Figure 13(b_2_)). The surface topographies of the PVPA-modified Ti6Al4V and the PTFE ball at the velocity of 24 mm/s can be seen in Figure 11(b_3_). No obvious wear can be seen. It can be considered that the PVPA coating can significantly reduce the dependence of surface friction properties on velocity.

The PVS coating physically adsorbed on Ti6Al4V can improve the bearing characteristics of the surface and reduce the dependence of the surface friction properties on velocity. For the polymer coating connected to the substrate by chemical bonding, the PVPA coating can maintain an ultra-low friction state in the load and velocity range studied, while the friction coefficient of the PAA-modified Ti6Al4V was higher than that of bare Ti6Al4V. The modification of the PAA coating has no obvious effect on the improvement of the surface bearing capacity and velocity dependence.

## 4. Conclusions

Three polymer coatings, namely PVS, PAA and PVPA, were prepared on Ti6Al4V using the method of self-assembly. The regulation mechanism of the special functional groups contained in the polymer molecular chains on the tribological properties of the modified surfaces was analyzed, mainly by means of experimental analysis and molecular simulation.

The functional groups can affect the binding method of the polymer coatings to Ti6Al4V, and the interactions between polymer molecular chains and the surrounding environment. The sodium sulfonate groups cause the PVS molecular chains to physically adsorb on the Ti6Al4V. The PVS coating can enhance the ability to attract water molecules through Na^+^ adsorbed on the PVS molecular chains, which can improve the tribological properties of Ti6Al4V. However, the anti-friction effect of the PVS coating cannot be durable in the long-term or with repeated usage. The carboxyl groups and phosphate groups contained in the PAA and PVPA molecular chains can form chemical bonds with the oxide layer on Ti6Al4V. However, the tribological properties of PAA-modified Ti6Al4V and PVPA-modified Ti6Al4V showed significant differences. Carboxyl groups in the PAA molecular chains cannot firmly bind to the substrate, and salt ions in the NaCl solution will promote the surface wear of PAA-modified Ti6Al4V in the friction process, resulting in a high friction coefficient. Furthermore, the phosphate group causes the polymer coating to firmly bind to the substrate, making it more attractive to salt ions and water molecules to form a hydrated layer on the coating surface, which can enable PVPA-modified Ti6Al4V to obtain an ultra-low friction coefficient and ultra-low wear. This study can provide effective guidance for the design of polymer coatings to produce modified metals.

## Figures and Tables

**Table 1:** The mass ratios of surface element of Ti6Al4V and polymer-modified Ti6Al4V surfaces after soaking in a NaCl solution.

**Elements**	Ti	Al	V	C	O
Ti6Al4V	86.55 ± 0.38	5.57 ± 0.06	3.42 ± 0.12	1.56 ± 0.11	2.91 ± 0.39
PVS	84.17 ± 0.24	5.48 ± 0.04	3.68 ± 0.09	3.67 ± 0.10	2.37 ± 0.24
PAA	1.54 ± 0.05	0 + 0.02	0 + 0.05	58.65 ± 0.17	22.58 ± 0.16
PVPA	1.6 ± 0.03	0 + 0.02	0 + 0.03	24.44 ± 0.20	27.01 ± 0.11
	S	P	Na	Cl	
Ti6Al4V	/	/	0 + 0.04	0 + 0.03	
PVS	0 + 0.03	/	0.36 ± 0.03	0.26 ± 0.02	
PAA	/	/	10.41 ± 0.06	6.82 ± 0.05	
PVPA	/	21.67 ± 0.08	18.03 ± 0.06	7.25 ± 0.04	

**Table 2:** The mass ratios of surface elements of wear marks.

**Elements**	Ti	Al	V	C	O
Ti6Al4V	65.18 ± 0.19	4.03 ± 0.04	2.74 ± 0.08	2.43 ± 0.14	1.61 ± 0.21
PVS	82.23 ± 0.23	5.35 ± 0.04	3.72 ± 0.08	3.67 ± 0.10	2.55 ± 0.23
PAA	75.80 ± 0.19	4.82 ± 0.03	3.41 ± 0.07	4.63 ± 0.11	1.78 ± 0.19
PVPA	52.95 ± 0.17	3.13 ± 0.03	1.88 ± 0.07	10.67 ± 0.11	14.10 ± 0.18
	S	P	Na	Cl	
Ti6Al4V	/	/	13.07 ± 0.07	10.94 ± 0.06	
PVS	0 + 0.03	/	1.44 ± 0.04	1.03 ± 0.02	
PAA	/	/	5.34 ± 0.04	4.23 ± 0.03	
PVPA	/	5.45 ± 0.03	7.46 ± 0.05	4.36 ± 0.03	

**Table 3:** Calculated interaction energies of the polymer chains with salt ions and the water molecule.

Polymer	Form	Interaction Energy (Kcal/mol)
PVS	PVS-Na^+^	−33.31
	PVS-Cl^−^	0.21
	PVS-H_2_O	1.08
PAA	PAA-Na^+^	−3.23
	PAA-Cl^−^	−1.77
	PAA-H_2_O	−8.90
PVPA	PVPA-Na^+^	−6.33
	PVPA-Cl^−^	−3.09
	PVPA-H_2_O	−14.91

## Data Availability

Not applicable.

## Supplementary Materials

The following supporting information can be downloaded at: https://www.mdpi.com/article/10.3390/polym15204060/s1, Figure S1: A long friction experiment with NaCl solution lubrication: (a) the average of friction coefficient for 4 hours; (b) the friction coefficient versus time; (c) wear morphologies of the bare Ti6Al4V and the PTFE ball; (d) wear morphologies of the PVS-modified Ti6Al4V and the PTFE ball; (e) wear morphologies of the PAA-modified Ti6Al4V and the PTFE ball; (f) wear morphologies of the PVPA-modified Ti6Al4V and the PTFE ball.; Figure S2: Tribological properties of the PVS-modified Ti6Al4V under dry friction experiment: (a) the average of friction coefficient; (b) wear morphologies of the PVS-modified Ti6Al4V and the PTFE ball for the first friction; (c) wear morphologies of the PVS-modified Ti6Al4V and the PTFE ball for the second friction; (d) wear morphologies of the PVS-modified Ti6Al4V and the PTFE ball for the third friction.
