# Supplementary material for: Regulation Mechanism of Special Functional Groups Contained in Polymer Molecular Chains on the Tribological Properties of Modified Ti6Al4V
Source: Polymers (Basel). 2023 Oct 12;15(20):4060. doi: 10.3390/polym15204060 (PMC10609861; doi:10.3390/polym15204060)
Supplement: Supplementary file 1 [file polymers-15-04060-s001.zip › polymers-2617494-supplementary.pdf]

Article

Supporting Information

Regulation Mechanism of Special Functional Groups Contained in Polymer Molecular Chains on the Tribological Properties of Modified Ti6Al4V

Mengmeng Liu [1], Jing Ni [1], Caixia Zhang [2,*], Lihui Wang [2], Yue Guo [1] and Zhifeng Liu [3,*]

[1] School of Mechanical Engineering, Hangzhou Dianzi University, Hangzhou 310018, China; 42895@hdu.edu.cn (M.L.); nj2000@hdu.edu.cn (J.N.); 222010095@hdu.edu.cn (Y.G.)

[2] Institute of Advanced Manufacturing and Intelligent Technology, Beijing University of Technology, Beijing 100124, China; wanglihui@emails.bjut.edu.cn

[3] Key Laboratory of CNC Equipment Reliability, Ministry of Education, Jilin University, Changchun 130012, China

* Correspondence: zhang-cx15@bjut.edu.cn (C.Z.); lzfjlu@jlu.edu.cn (Z.L.)

Citation: Liu, M.; Ni, J.; Zhang, C.; Wang, L.; Guo, Y.; Liu, Z. Regulation Mechanism of Special Functional Groups Contained in Polymer Molecular Chains on the Tribological Properties of Modified Ti6Al4V. *Polymers* **2023**, *15*, x. https://doi.org/10.3390/xxxxx

Academic Editor: Zilu Wang

Received: 2 September 2023
Revised: 1 October 2023
Accepted: 5 October 2023
Published: 11 October 2023

Copyright: © 2023 by the authors. Submitted for possible open access publication under the terms and conditions of the Creative Commons Attribution (CC BY) license (https://creativecommons.org/licenses/by/4.0/).

S1. A long friction experiment with NaCl solution lubrication

The friction experiments were performed at room temperature in the reciprocating mode with an initial load of 2.5 N. The sliding speed was 12 mm/s. The bottom friction pair were the bare Ti6Al4V, the PVS-modified Ti6Al4V, the PAA-modified Ti6Al4V and the PVPA-modified Ti6Al4V, successively. The upper friction pairs were PTFE balls. The NaCl solution with a concentration of 0.5 mol/l was used as a lubricant. During the four hours of friction, the friction coefficients of the bare Ti6Al4V, the PVS-modified Ti6Al4V, the PAA-modified Ti6Al4V and the PVPA-modified Ti6Al4V were 0.0753 ± 0.0023 0.0737 ± 0.0024, 0.0674 ± 0.0070 and 0.0080 ± 0.0020, respectively, as shown in Figure S1(a). Compared with the friction time of 30 minutes (Figure 6), the friction coefficient of the bare Ti6Al4V surface and the wear marks of friction pairs were significantly increased, as shown in Figure S1 (c) and Figure 6(c). The friction coefficients of PVS-modified Ti6Al4V, PAA-modified Ti6Al4V and PVPA-modified Ti6Al4V surfaces and the corresponding wears of friction pairs also increase and intensify with the increase of friction time, as shown in Figure S1(c), (d), (e) and Figures 6(c), (d), (e). However, the PVPA-modified Ti6Al4V surface is always in an ultra-low friction state during the friction process, as shown in Figure S1 (b). The trends of friction coefficient of PAA-modified Ti6Al4V surface and the wear marks of friction pairs over time are also smaller than that of the PVS-modified Ti6Al4V. Therefore, it can be considered that PVPA is the most durable of the three modified coatings, followed by PAA and PVS.

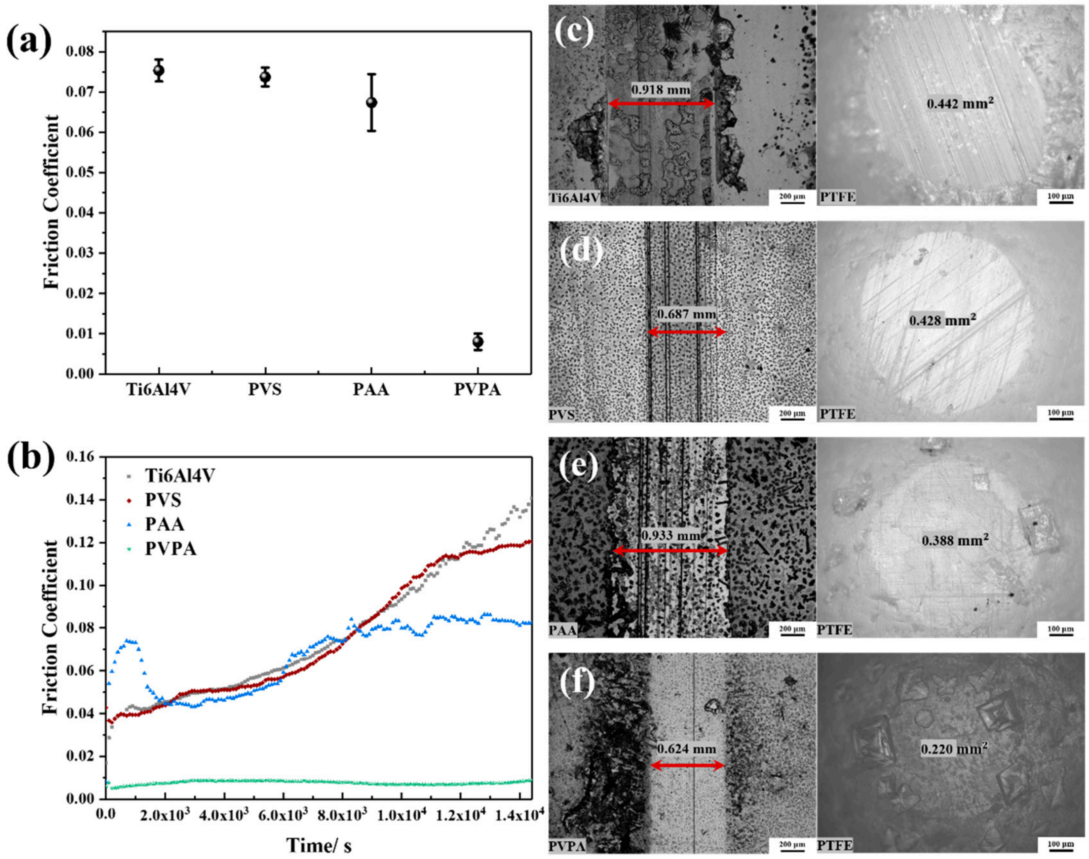

Figure S1. A long friction experiment with NaCl solution lubrication: (a) the average of friction coefficient for 4 hours; (b) the friction coefficient versus time; (c) wear morphologies of the bare Ti6Al4V and the PTFE ball; (d) wear morphologies of the PVS-modified Ti6Al4V and the PTFE ball; (e) wear morphologies of the PAA-modified Ti6Al4V and the PTFE ball; (f) wear morphologies of the PVPA-modified Ti6Al4V and the PTFE ball.

S2. Dry friction experiment

The first friction experiment was performed at room temperature in the reciprocating mode with an initial load of 2.5 N. The sliding speed was 12 mm/s. The bottom friction pair were the PVS-modified Ti6Al4V. The upper friction pair was PTFE ball. The NaCl solution was used as a lubricant. After the end of the friction experiment, the PVS-modified Ti6Al4V and the PTFE ball were taken out and dried to detect the wear marks. The results can be seen in Figure S2(b). With other parameters unchanged, the PVS-modified Ti6Al4V sample after the first friction test and a new PTFE ball were used for the second friction test. The sliding area of the second friction test was guaranteed to be coincident with that of the first friction test. Wear marks of the PVS-modified Ti6Al4V and the PTFE ball after the second friction test are shown in Figure S2 (c). The third friction test was conducted in the same way, and the wear marks of friction pairs are shown in Figure S3 (c). The time of the three friction experiments are all 30 minutes, and the change trends of friction coefficients with time can be seen in Figure S2 (a). The average of friction coefficients corresponding to the first, the second and the third friction tests were 0.0379, 0.0434 and 0.0513 respectively, showing a gradually increasing trend. After the first, the second and the third friction tests, the wear marks of PVS-modified Ti6Al4V showed a gradually increasing trend. The corresponding PTFE friction pair also has the same trend of increasing wear.

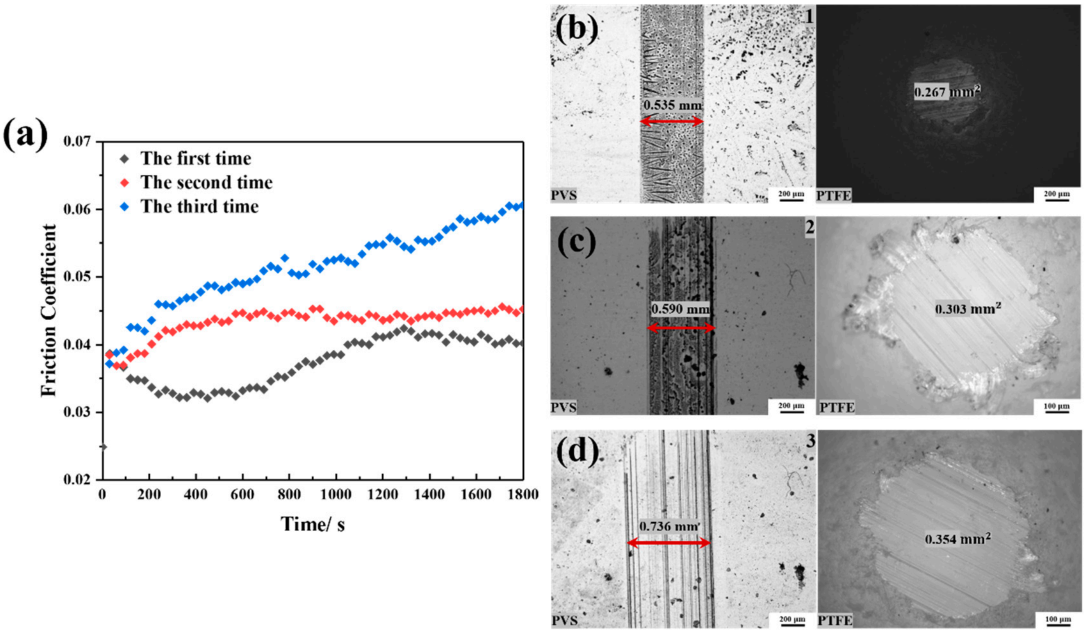

Figure S2. Tribological properties of the PVS-modified Ti6Al4V under dry friction experiment: (a) the average of friction coefficient; (b) wear morphologies of the PVS-modified Ti6Al4V and the PTFE ball for the first friction; (c) wear morphologies of the PVS-modified Ti6Al4V and the PTFE ball for the second friction; (d) wear morphologies of the PVS-modified Ti6Al4V and the PTFE ball for the third friction.
